# Stretchable, Highly Durable Ternary Nanocomposite Strain Sensor for Structural Health Monitoring of Flexible Aircraft

**DOI:** 10.3390/s17112677

**Published:** 2017-11-20

**Authors:** Feng Yin, Dong Ye, Chen Zhu, Lei Qiu, YongAn Huang

**Affiliations:** 1 State Key Lab of Digital Manufacturing Equipment and Technology, School of Mechanical Science and Engineering, Huazhong University of Science and Technology, Wuhan 430074, China; yinfeng@hust.edu.cn (F.Y.); yedong@hust.edu.cn (D.Y.); zhuchen@hust.edu.cn (C.Z.); 2 Flexible Electronics Research Center, Huazhong University of Science and Technology, Wuhan 430074, China; 3 Research Center of Structural Health Monitoring and Prognosis, State Key Lab of Mechanics and Control of Mechanical Structures, Nanjing University of Aeronautics and Astronautics, Nanjing 210016, China; lei.qiu@nuaa.edu.cn

**Keywords:** strain sensor, conductive nanocomposite, structural health monitoring, aerostat

## Abstract

Harmonious developments of electrical and mechanical performances are crucial for stretchable sensors in structural health monitoring (SHM) of flexible aircraft such as aerostats and morphing aircrafts. In this study, we prepared a highly durable ternary conductive nanocomposite made of polydimethylsiloxane (PDMS), carbon black (CB) and multi-walled carbon nanotubes (MWCNTs) to fabricate stretchable strain sensors. The nanocomposite has excellent electrical and mechanical properties by intensively optimizing the weight percentage of conducting fillers as well as the ratio of PDMS pre-polymer and curing agent. It was found that the nanocomposite with homogeneous hybrid filler of 1.75 wt % CB and 3 wt % MWCNTs exhibits a highly strain sensitive characteristics of good linearity, high gauge factor (GF ~ 12.25) and excellent durability over 10^5^ stretching-releasing cycles under a tensile strain up to 25% when the PDMS was prepared at the ratio of 12.5:1. A strain measurement of crack detection for the aerostats surface was also employed, demonstrating a great potential of such ternary nanocomposite used as stretchable strain sensor in SHM.

## 1. Introduction

Strain sensors, tightly mounted on/in measured objects, have been widely used in aerospace, wearable electronics, civil engineering, human-machine interface, etc., to monitor and record the healthy condition. Despite their attractive features, conventional strain sensors, such as foil strain gauge, Fiber Bragg Grating and semiconductor strain sensor, show some limitations in terms of low gauge factor (GF ~ 2.2), weak deformation ability and small measurement range (<2%) that hinder the utilization in stretching conditions [1]. Especially nowadays, in flexible and complex systems, such as morphing aircraft, inflated spacecraft and aerostats, the structure deformation usually are large (>10%) and damage or crack that can occur anywhere over the structure is difficult to detect [2]. Moreover, the strain sensor should also be able to conformably attach onto irregular non-planar surface and can deform with the flexible structures without deteriorating the sensing function. Apparently, the conventional strain sensors often are not practical in such conditions.

Various attempts have been made to obtain such desirable sensors. Nanoscale metal films [3] and metal films with buckling/serpentine/self-similar geometries on elastomer substrates [4,5,6,7]. However, these sensors are satisfied with the requirement of large deformation but are not durable and stable when are subjected to the dynamic load. Recent researches on stretchable strain sensors have focused on the use of nanoscale carbon materials such as carbon black (CB), carbon nanotubes (CNTs) and graphene, etc. to overcome the challenges associated with structural health monitoring (SHM) in flexible materials [8,9,10,11]. Nanoscale carbon materials can be used as strain-sensing materials either individually or as conductive fillers in soft polymer. Among these materials, CNTs, which is an ideal candidate for strain sensor in SHM, have attracted considerable attention because of their superior mechanical properties as well as excellent conductivity and high aspect ratio [12]. The groups of Chou [13,14] and Peijs [15,16] have investigated the application of CNTs in in-situ detection and progress of damage in glass fiber-reinforced polymers (GFRPs). Their studies revealed that the embedded CNTs fibers in GFRPs provide the possibility of in-service health monitoring and multiple locations sensing without complicated instruments. Yet, the limitation of such method is the destructive testing and is not available for large deformation. Other studies focused on piezoresistive nanocomposites made by homogenously dispersing CNTs into a flexible polymer matrix through the mechanical, physical or chemical methods [17,18,19,20,21]. These kinds of nanocomposites not only remain the remarkable durability and stretchability of the polymer matrix, but also possess the electrical conductivity of conducting fillers. Furthermore, it can be mounted on measured structures to sense the healthy condition via the resistance-strain characteristic.

Currently, the most of conductive nanocomposites based CNTs are binary composites. The binary nanocomposites with simple and low-cost preparation process more easily obtain the satisfactory electrical conductivity, but it may result in the high percolation threshold and poor mechanical properties. To further reduce the percolation threshold of composites, the ternary nanocomposites have been developed because of the benefits in decreasing the fillers content of CNTs by adding the secondary nanofiller via the synergistic effect of hybrid fillers [22,23]. Yet, the determination on the weight percentage of conducting fillers just relies on the electrical conductivity or additional functionalities (such as barrier properties and fire retardancy [24,25]) of ternary nanocomposites without consideration the applications in strain sensing.

In this paper, we introduced a highly durable ternary conductive nanocomposite consisting of polydimethylsiloxane (PDMS), CB nanoparticles and multi-walled carbon nanotubes (MWCNTs) via optimizing the process parameters including weight percentage of conducting fillers and the ratio of PDMS pre-polymer and curing agent in terms of electrical conductivity and strain sensitivity of nanocomposite. The ternary nanocomposite is easy to be fabricated into thin-film devices by screen printing and transfer printing. In this way we realized a stretchable strain sensor that exhibited remarkable durability (over 10^5^ cycles at 25% strain), high sensitivity (GF ~ 12.15) as well as good linearity and reproducibility. These superior properties allow the prepared nanocomposite to be used to crack detection in SHM, as was demonstrated by attaching the strain sensors onto an aerostat structure in contact to record the change of resistance.

## 2. Materials and Methods

### 2.1. Preparation of Ternary Conductive Nanocomposites

The MWCNTs (PMW307), with outer diameter of ~15 nm and an average length of ~45 μm, were purchased from Nachen Technology Company (Beijing, China). The aspect ratio varying from 100 to 350 also characterized the MWCNTs morphology. The conductive spherical CB (VXC-72R, Cabot, Boston, MA, USA) nanoparticle with an average diameter of 45 nm was served as another hybrid conducting filler. Viscoelastic material, PDMS (Sylgard 184 Kit A, Dow Corning, Midland, MI, USA), was utilized as the matrix material because of its low mechanical impedance, good conformal ability and easy fabrication [26].

**Figure 1:**
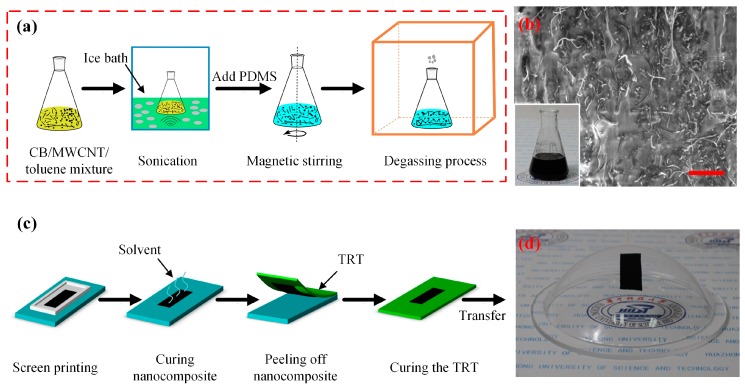
Schematic diagram of ternary nanocomposite preparation, fabrication and transfer printing process. (**a**) Synthesis process of nanocomposite; (**b**) SEM image of prepared nanocomposite (inset: liquid nanocomposite before curing), the scale bar is 5 μm; (**c**) Fabrication and transfer printing process of ternary nanocomposite thin film; (**d**) Image of nanocomposite specimen was transferred to a complicated surface.

The ternary conductive nanocomposite (CB/MWCNTs/PDMS) was synthesized by homogeneously dispersing the conducting CB nanoparticles and MWCNTs into the prepared PDMS matrix, as schematically shown in Figure 1a. Specifically, the first step was to add certain weight fractions of the conducting fillers into the volatile solvent. The toluene (Sinopharm Chemical Reagent Co., Ltd., Shanghai, China) was selected as the solvent here because of its significant performance in scattering the CB nanoparticles and MWCNTs. The mixture of CB/MWCNTs/toluene material was then decentralized using an ultrasonic cleaner (KQ-600KDE, Kunshan Ultrasonic Instrument Co., Ltd., Kunshan, China) with optimized power of 40 W for 50 min in an ice bath. Subsequently, the prepared PDMS was poured into the beaker flask and further disaggregated with a magnetic stirrer (HJ-4A, Jintan Youlian Instrument Research Institute, Jintan, China) for 2.5 h to obtain a homogeneous nanocomposite. Finally, degassing was carried out in a vacuum chamber (ZK-6050A, Wuhan Aopusen Test Equipment Co., Ltd., Wuhan, China) to remove the gas bubbles in the liquid nanocomposite. Additionally, the scanning electron microscope (SEM, Helios NanoLab G3, FEI Co., Hillsboro, WI, USA) was employed to observe the dispersion and mosaic results of ternary conductive nanocomposite. The SEM image shows that the CB and MWCNTs were quite homogenously embedded into the PDMS matrix among the prepared samples (Figure 1b).

### 2.2. Fabrication and Transfer of Ternary Conductive Nanocomposites Thin Film

A thin film of the ternary conductive nanocomposite was adopted to characterize the electrical and mechanical properties. A simply rapid fabrication and transfer method was presented by combination of screen printing and transfer printing, as illustrated systematically in Figure 1c. Firstly, a mask with special pattern was fixed on the cleaned glass substrate and then the prepared uncured ternary nanocomposite was deposited on it. For this, the pattern was cut into rectangular dimensions (15 mm × 8 mm). The next step was to cure the composites at 80 °C for 6 h at the vacuum chamber to wipe out the rest solvent for a high durability and low Young’s modulus. Subsequently, the cured conductive thin film was carefully peeled off using a thermally released tape (TRT, No. 3195MS, Nitto, Osaka, Japan). Here the stripped nanocomposite cured on a hotplate at 130 °C for 3 min and later it could be transferred onto the other surface area in contact. As shown in Figure 1d, the conductive thin film could be successfully transferred onto flat surface and even complex surface, implying the potential applications in conformal bonding on arbitrary surface.

### 2.3. Characterization

The electrical conductivity of ternary nanocomposite was measured with a two-probe method at 22 °C in clean room using a precision semiconductor characterization system (SCS, Keithley4200, Tektronix, Cleveland, OH, USA). The specimens were prepared by rapid fabrication and transfer method and grinded the rough surface into a thickness of 0.5 mm according to the ASTM D3039 specification. In order to reduce the contact resistance between the sample and the electrodes, a double-sided conductive copper foil with a thickness of 0.04 mm was attached to the surface and then the sample was clamped on two sides with two alligator clips. The measured conductivity *σ* could be calculated by the following equation:

*σ* = *L*/(*RWT*), (1)

where *R* is the resistance of conductive nanocomposites sample, *W*, *T* and *L* are the width, thickness and length of the nanocomposites specimen, respectively.

The specimen tests of strain-resistivity characteristics under cyclic loading/unloading were carried out at a constant speed of 2 mm/s under the same strain using a homemade automatic re-stretching machine, which connects with the SCS via coaxial cables so as to detect and record the experimental data in real time. Tensile strength was characterized through a one-way electronic universal testing machine (9500, Instron, Boston, MA, USA) based on ASTM D638-98 method. The transverse sensitivity was measured by installed four specimens in an equal intensity beam. Importantly, it needs to be noticed that electrical conductivity, resistance and tensile strength of each sample were obtained based on at least three specimens per sample. The average values and standard deviations were presented.

## 3. Results and Discussion

### 3.1. Optimization of the Processing Parameters

#### 3.1.1. The Weight Percentage of Conducting Fillers

In terms of the mechanical and electrical properties of ternary nanocomposite, the weight percentage of conducting fillers was emphatically investigated here. Thus a creatively improved method on the basis of electrical conductivity and strain sensitivity was presented to optimize the weight percentage of fillers so as to obtain the ternary conductive nanocomposite with excellent GF. The key strategy was to ensure one of filler content from the electrical conductivity and another hinged on the strain sensitivity.

**Figure 2:**
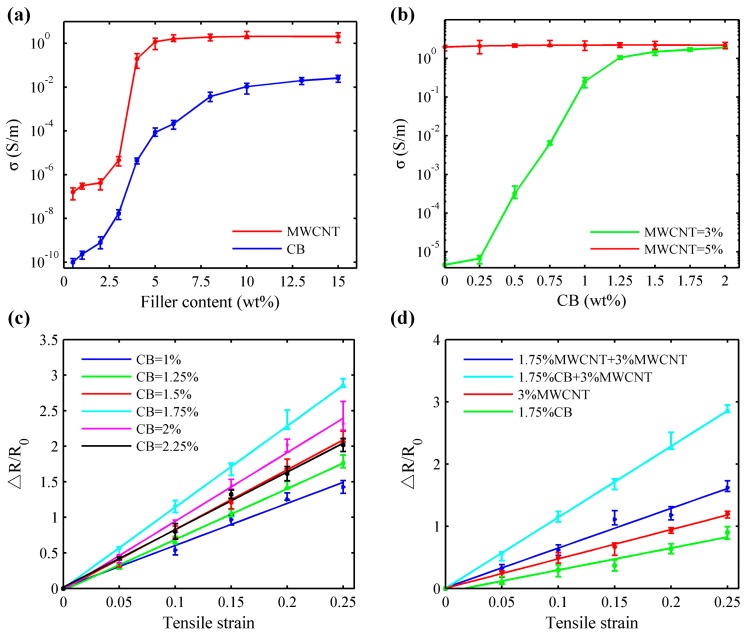
The electrical conductivity of nanocomposites with different fillers content and the results of resistance change ratio (Δ*R*/*R*_0_) under varying tensile strain. (**a**) Conductivity of binary nanocomposites containing CB or MWCNT (inset: Amplified image of CB/PDMS nanocomposite conductivity); (**b**) Conductivity of ternary nanocomposites with variable CB contents when weight percentage of MWCNT was fixed as 3% and 5%, respectively; (**c**) The results of resistance change ratio with the fixed 3 wt % MWCNT and variable CB content under the applied tensile strain of up to 25%; (**d**) The function of resistance change ratio with the special combination of MWCNT and CB contents.

Figure 2a shows electrical conductivity of the binary CB/PDMS and MWCNTs/PDMS nanocomposites as a function of the filler content. It can be found that the incorporation of MWCNTs alone increased the conductivity of MWCNTs/PDMS composites by almost 7 orders of magnitude, from 1.6 × 10^−7^ to 2.2 S/m when the weight percentage of MWCNTs was increased from 0% to 15% with a percolation threshold at about 5% (red curve). But for the same range of filler content, the composite comprised of CB nanoparticles and matrix PDMS (blue curve) exhibits a higher percolation threshold (nearly 15%). Additionally, compared with the prepared MWCNTs/PDMS nanocomposite, the saturated conductivity of binary CB/PDMS nanocomposite (~2.58 × 10^−2^ S/m) is significantly lower almost 2 orders of magnitude because of the point-to-point contacts between CB nanoparticles caused by poor performance in aspect ratio [27], which are corresponding to the static percolation theory [28]. This means that the content of MWCNTs plays a critical role in forming the conducting network of whole ternary nanocomposite.

Figure 2a shows that the electrical conductivity of conducting fillers changes sharply when the content of MWCNTs ranges from 3% to 5%. On account of nanocomposite electrical conductivity, further investigations were carried out to find out the optimal content of MWCNTs by blending a variable content of CB nanoparticles with the fixed MWCNTs content of 3% and 5%, which corresponds to those below and at the percolation threshold content of MWCNTs. The results shown in Figure 2b indicated that there was a remarkable increase in electrical conductivity by almost 5 orders of magnitude with an extra addition of about 1.75 wt % CB nanoparticles. The percolation threshold of the composites (MWCNTs/PDMS) also decreases with the addition of secondary nano-filler (CB). The possible reason is the synergistic effect of incorporating secondary conducting fillers with different shapes and aspect ratios in nanocomposite [23,29]. Additionally, there is little contribution to the enhancement of nanocomposite conductivity when extra CB content was added into the 5 wt % MWCNTs, indicating that the synergistic effect is non-effective once the conducting networks are basically formed. Therefore, 3 wt % was selected as the filling content of MWCNTs for the ternary nanocomposite.

Strain sensitivity is regarded as a key factor to evaluate the measurement performance of stretchable strain sensors. Thus it was exploited in here to obtain the optimal CB content. Figure 2c shows the results of resistance change ratio (ΔR/R_0_) of the specimens with the fixed 3 wt % MWCNTs and variable CB content under the applied tensile strain of up to 25%. The measured points were fitted with linear curves by least squares method, the slope of which represents the GF of a sample. It shows that the slopes of fitting lines have an upward trend until the incremental content of CB nanoparticles is greater than 1.75 wt %. Once the content of hybrid CB is over this value, the slopes will have a slight bit decline. This indicates that adding 1.75 wt % CB nanoparticles into MWCNTs/PDMS nanocomposites can exhibit optimal strain sensitivity. The possible reason involved in the above phenomenon is the enhancement of tunneling effect and improvement of electrical resistance caused by decreasing the contacting distance between the MWCNTs and CB nanoparticles due to the synergistic effect of different conducting fillers when the containing CB content is less than 1.75%. Once the weight percentage is over the 1.75%, the electron tunneling will be redundant and overlapped. Thus the resistance change of nanocomposite becomes smaller when applied the same strain range (25%), resulting in the decline of strain sensitivity. Figure 2d is a supplementary explanation to Figure 2c, where the combination of 1.75 wt % CB + 3 wt % MWCNTs has the higher GF than that of the mixture of 1.75 wt % MWCNTs + 3 wt % MWCNTs, only 1.75 wt % CB or individually 3 wt % MWCNTs. It declaims that adding an extra of CB nanoparticles into MWCNTs/PDMS nanocomposites can improve the nanocomposite strain sensitivity. Therefore, 1.75 wt % CB was served as another hybrid filler content in the developed ternary nanocomposite.

#### 3.1.2. The Ratio of PDMS Pre-Polymer and Curing Agent

As expected, the ratio of pre-polymer and curing agent plays a critical role in formation and characteristics of PDMS and it also affects the mechanical performance of the prepared ternary nanocomposite because the main component of pre-polymer is poly(dimethyl-methylvinylsiloxane) and the component of curing agent is the poly(dimethyl-methylhydrogenosiloxane) with the vinyl side chain, which will occur the hydrogenated silanization reaction and form three dimensional net structure when is mixed with the different ratio [30]. For this, further experiments were carried out to investigate the influence of different ratio of PDMS pre-polymer and curing agent to tensile strength of nanocomposite so as to obtain the optimal ratio. Table 1 shows the results of electrical conductivity and tensile strength of fabricated nanocomposites specimens under the variable ratio of 5:1, 7.5:1, 10:1, 12.5:1, 15:1, 17.5:1 and 19:1 with a fixed hybrid filler of 1.75 wt % CB and 3 wt % MWCNTs. Few changes in electrical conductivity of the nanocomposite reveals that the conductivity only depends on the conducting network formed by the hybrid fillers rather than affected by the PDMS matrix. In contrast, the changes of ratios have remarkable influence on nanocomposite tensile strength. As demonstrated in the Table 1, the nanocomposite tensile strength exhibits the maximum value 7.3 MPa at the ratio of 12.5:1, while it shows the poor performance at the 5:1 and 19.5:1. That is because the pre-polymer and curing agent did not completely react, resulting in the decrease of nanocomposite tensile strength. Therefore, it can be determined that the ratio of PDMS pre-polymer and curing agent at 12.5:1 is the optimal choice in this study.

### 3.2. Properties of Fabricated Ternary Nanocomposite

#### 3.2.1. Elastic Strain Performances

When the prepared nanocomposites samples are subjected to an external tensile load, a change in resistance of specimens will happen as a result of recombination of the conductive network formed by conducting hybrid fillers under stretchable deformation. This performance is greatly desirable for applying as stretchable strain sensors, which can be widely used in SHM systems to monitor and record the healthy condition of a structural element.

**Figure 3:**
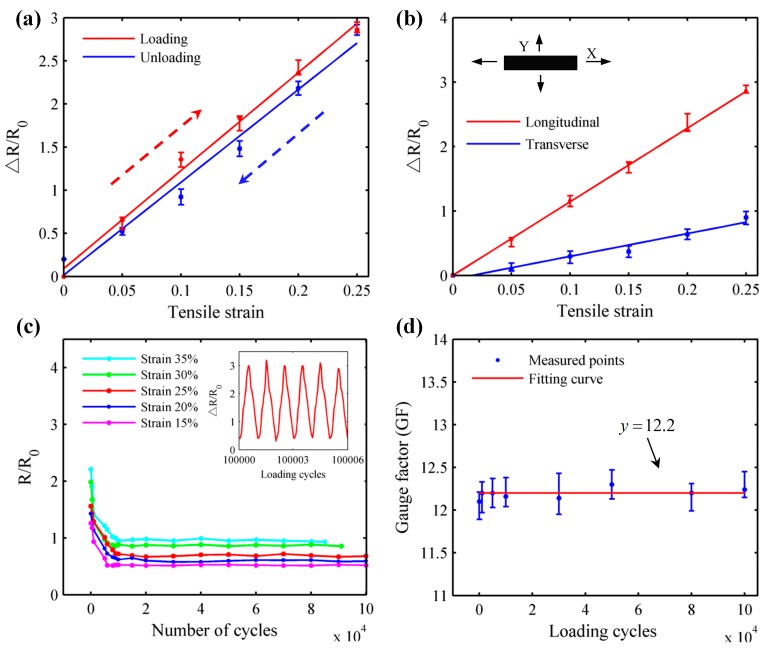
Elastic strain performances of fabricated nanocomposite specimens. (**a**) The sensor characteristics curves in single loading/unloading process; (**b**) The sensor response curves in both longitudinal (*X* axis) and transverse (*Y* axis) directions; (**c**) The resistance ratio of nanocomposite specimens under cyclic loading and unloading with the varying tensile strain (inset: the Δ*R*/*R*_0_ of nanocomposite with tensile strain of up to 25%); (**d**) The GF response curve of specimen as a function of loading cycles.

The characteristics of the nanocomposites samples are plotted in Figure 3a. A tensile strain of up to 25% was applied along the longer axis of the specimen. The response of nanocomposite in resistance change ratio vs. tensile strain is nearly linear during the whole loading and unloading process. This means that the prepared nanocomposite has the great potential application used as strain gauge and it can overcome the strain limit of commercial foil strain gauges. However, a small viscous hysteresis can be observed from the Figure 3a and there is also a slight increase in resistance change ratio at the recovery process. The factors are the unavoidable characters of fabricated nanocomposite caused by the recombination of electrical hybrid fillers and slow creep of viscoelastic matrix.

An ideal strain gauge is one measuring the strain only along a desirable direction [31]. Actually, it is inevitable for strain sensor to encounter sensitivity loss due to the transverse influence of the grid geometry and intrinsic property in strain sensing element. Thus further tests were carried out to explore the transverse sensitivity of the prepared nanocomposite. The sensor response curves of transverse sensitivity are depicted in Figure 3b. Apparently, the GFs of elastic strain sensor along the longitudinal and transverse directions are basically constant during the whole strain range (25%) and their numerical values are about 12 and 3.1, respectively. Thus the transverse sensitivity can be calculated by the following equation:

*C = K_y_/K_x_* (2)

where *C* is the transverse sensitivity of fabricated elastic strain, *K_x_* and *K_y_* are the GF of the strain along the longitudinal and transverse directions, respectively. Based on the aforementioned equation, the transverse sensitivity of strain sensor is approximate 26%, which exhibits better results in comparison with the published literature [32,33]. This excellent performance can not only help to detect the crack of the structure, but also determine the direction of the applied load. Though the strain sensors could not catch up with the performance of the foil strain gauges in transverse sensitivity, an enhancement could be realized by external special processing circuit in the future work.

Figure 3c shows the resistance ratio of nanocomposite specimens under stretching-releasing cycles with applied tensile strain of 15%, 20%, 25%, 30% and 35%, respectively. Obviously, the resistances of the nanocomposites tend to be stable after a remarkable decrease with thousands of cycles in tension. The possible reasons involved in the above phenomenon are the relative slippage of aggregated conductive fillers when are subjected the external load, resulting in forming the new conducting pathways. Yet, the stretchable cycles could be decline with the increase of applied strain due to the cumulative fatigue damage. For example, the durability in strain of 35% is far shorter than the performance in 25%, which will break up at about 82,000 cycles. The inset in Figure 3c shows that the specimen with application of 25% strain keeps good reproducibility and superior durability over 10^5^ cycles, indicating great potential as strain gauge to detect the crack caused by the dynamic loadings. Besides, the GF response curve of specimen after different cycles is demonstrated (Figure 3d). The GF of ~12.25 keeps almost constant during the whole stretching/releasing tests, which is much greater than the foil strain gauge with a low GF of ~2.2.

#### 3.2.2. Positive Temperature Coefficient Effect

**Figure 4:**
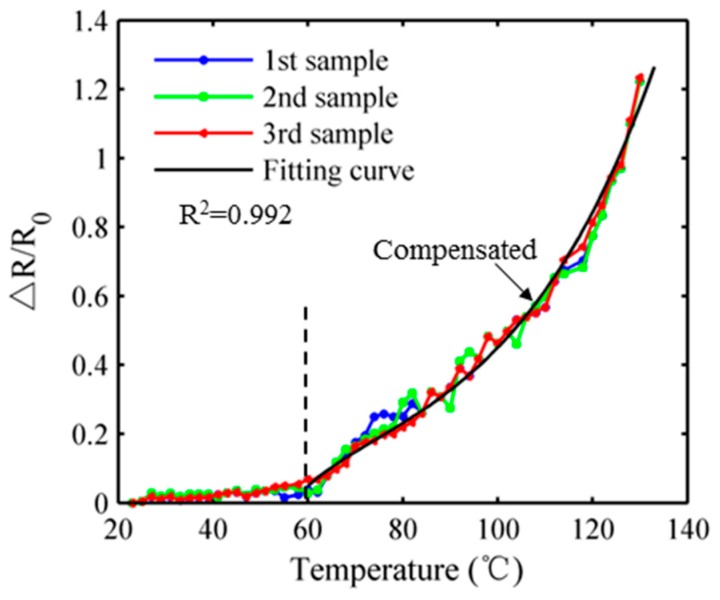
The temperature-resistivity characteristics of prepared ternary nanocomposites.

Temperature sensitivity is a critical factor for behavior of strain sensor in practical application [34]. Thus the temperature-resistivity characteristic of prepared ternary nanocomposite ranged from 20 °C to 130 °C was further explored, as illustrated in Figure 4. During a low temperature range from 20 °C to 60 °C, it exhibits negligible positive temperature coefficient effect on the performance of strain sensor. However, when the temperature increases over 60 °C, an obvious increase in the resistance possibly originated from the breakdown of conducting network after thermal expansion or the flow of PDMS matrix that acts like a viscous liquid under high temperature. The direct correlation between the resistance change ratio and temperature can also be seen in Figure 4. An exponential function was used to fit the measured points when the temperature ranges from 60 °C to 130 °C and it reflects good correlation (R^2^ = 0.992), implying that a temperature compensated algorithm can be proposed to remove the temperature drifting error by simultaneously using the measured temperature data.

### 3.3. Ternary Nanocomposite Mechanism Working as a Strain Sensor

**Figure 5:**
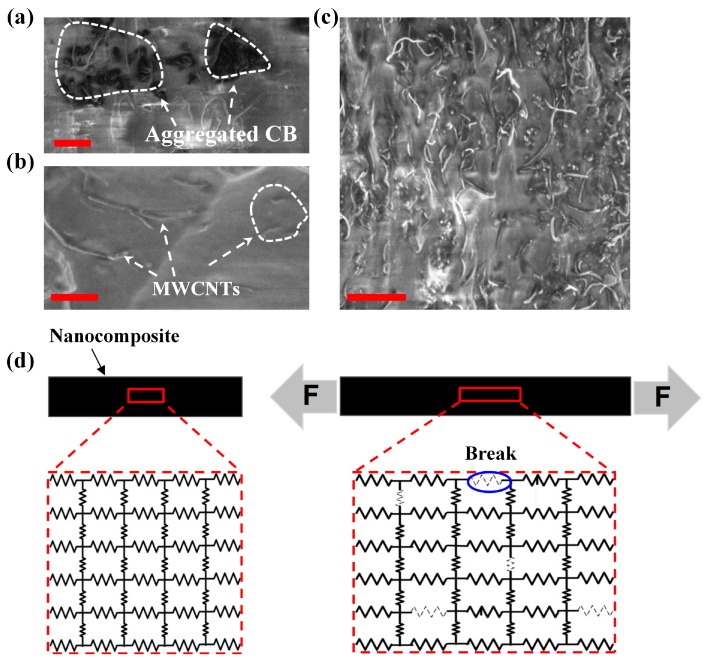
Nanocomposite mechanism working as a strain sensor. (**a**) SEM image of 1.75 wt % CB distributed in PDMS, the scale bar is 100 nm; (**b**) SEM image of 3 wt % MWCNT distributed in PDMS, the scale bar is 3 μm; (**c**) SEM image of hybrid fillers CB (1.75 wt %) and MWCNT (3 wt %) distributed in PDMS, the scale bar is 5 μm; (**d**) Schematic diagram showing the resistance change of nanocomposite under tensile strain.

To support the aforementioned excellent electrical and mechanical performances as strain sensor, the conducting and strain mechanism of ternary nanocomposite were schematically demonstrated in Figure 5. Figure 5a–c shows the SEM images of nanocomposite containing 1.75 wt % CB, 3 wt % MWCNTs and 1.75 wt % CB + 3 wt % MWCNTs, respectively. It is quite clear that the CB nanoparticles are scattered randomly with the morphology of individual or aggregation (Figure 5a) as well as the distribution of the MWCNTs (Figure 5b), but the global conducting networks in the binary nanocomposites system are not formed between them because of the insufficient filler content (note that the percolation threshold of CB and MWCNTs in PDMS are 15 wt % and 5 wt %, respectively). Yet, once these MWCNTs and CB particles are incorporated together, as shown in Figure 5c, the CB particles effectively link the gaps present between the unconnected MWCNTs because of the synergetic effect, leading to the enhancement of tunneling effect so as to form the global new conducting pathways in the nanocomposite [23].

Actually, at the nano-level, the conducting network structure of fabricated ternary nanocomposite is very complicated due to the crisscross hybrid fillers, but it can be equaled as a complex parallel circuit from the internal essence of the conducting network (Figure 5d). The resistors are representative of different conducting fillers. When a uniaxial tensile strain is applied to the nanocomposite, as shown in Figure 5d, the overall resistance will rise because of the breakage of contact points and widening of intertubular distances. Similarly, when nanocomposite is relaxed, conducting paths are restored; therefore, resistance drops along with decreasing strain.

### 3.4. Application as Strain Sensor for Crack Detection in SHM

**Figure 6:**
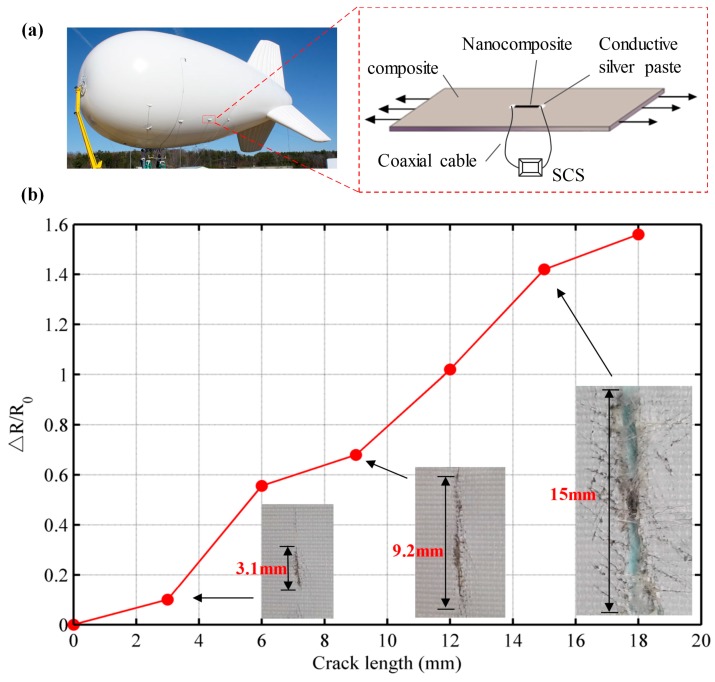
(**a**) Crack detection tests of aerostats with the prepared nanocomposite strain sensor; (**b**) The resistance change ratio of strain sensor under crack propagation (inset: photos of real cracks on the measured material).

The suspension location (left graph of Figure 6a) of aerostat is easy to fracture because of the high stress concentration and high-frequency oscillation caused by the wind. Once occurs the crack, it needs to repair the structure immediately otherwise the crack will augment and lead to the air leakage. Yet, it is difficult to detect the crack with the conventional strain sensors because the aerostat material is highly flexible (over 10%) and the measured area is large. More importantly, crack can occur anywhere over the composite structure. Thus it is of great significance to monitor the suspension location of aerostat in time with the prepared highly durable nanocomposite strain sensor. In this section, the prepared ternary conductive nanocomposite was used as stretchable strain sensor to detect crack on an aerostat composite when it was subjected to the external load. As depicted in Figure 6a, the nanocomposite thin film prepared based on the rapid fabrication and transfer method had been cut at the required dimensions (10 mm × 5 mm) and was polished on both sides into a thickness of 0.5 mm as the strain sensors. Then the strain sensors were placed in the middle of each testing aerostat composite (60 mm × 30 mm) via an epoxy adhesive (706, Xichen Co., Ltd., Changzhou, China). The testing aerostat composite is a flexible material with 12.6% elongation at break. Each strain sensor was covered with conductive silver paste on both sides to create a mean of “connector” to the material surface, which could attach the coaxial cable to measure and record the resistance during testing process through the SCS. The conductive silver paste was allowed to fully cure for 24 h at 25 °C. Subsequently, each specimen was tested on a one-way electronic universal testing machine under the varying loads to mimic the stretching process of aerostat material at the suspension location.

After the above operation, the resistance between the two ends of each stretchable strain sensor was measured and recorded for each testing specimen separately. Figure 6b shows the result of resistance change ratio of the strain sensor under crack progression. The deterioration caused by cross-sectional damage or a crack can be detected using the electrical property of the strain sensor because the nanocomposite thin film is sensitive to the change of resistance when the crack augments. The strength of the measured structure changes due to crack, which in turn influences the electrical performance of the strain sensor. The inset photos in Figure 6b shows the real cracks on measured aeronautical composite, implying that the normalized crack size can be described by the resistance change ratio of stretchable strain sensor. During the gradually augmentation of the crack, the resistance of strain sensor is increased until the structure totally fractures. As expected, the ternary nanocomposite strain sensor has the excellent performance to monitor the crack in SHM.

## 4. Conclusions

In summary, a highly durable ternary conductive nanocomposite (CB/MWCNTs/PDMS) with excellent elastic strain performance was developed and fabricated as stretchable strain sensor. After an intensive experimental optimization on the weight percentage of fillers content as well as the ratio of PDMS pre-polymer and curing agent, we found that a nanocomposite with hybrid fillers of 1.75 wt % CB and 3 wt % MWCNTs dispersed in PDMS matrix at the ratio of 12.5:1 exhibited distinctive electrical and mechanical behaviors. Strain sensors made from a thin film of such conductive nanocomposite presented a good linearity (in the strain range of 25%), high GF (~12.25), superior durability and reproducibility (over 10^5^ cycles) under tensile tests. In addition, the nanocomposite mechanism as strain sensor was further discussed, revealing that the superior performance probably is originated from the synergistic effect of conducting fillers in electrical conductivity. The stretchable strain sensor based on the prepared ternary nanocomposite was also demonstrated good performance in monitoring the occurrence and propagation of crack, which indicates the huge applications in SHM of flexible system such as morphing aircraft, inflated spacecraft and aerostats.

## Figures and Tables

**Table 1:** Electrical and mechanical properties of fabricated nanocomposites under variable ratios.

RPC	5:1	7.5:1	10:1	12.5:1	15:1	17.5:1	19:1
Conductivity (S/m)	2.12	1.99	2.15	2.18	2.14	2.15	1.96
Tensile Strength (MPa)	3.2	4.9	6.4	7.3	5.9	4. 6	2.1

RPC: ratio of pre-polymer and curing agent in PDMS.
